# Enrichment with Lucerne Hay Improves Sow Maternal Behaviour and Improves Piglet Survival

**DOI:** 10.3390/ani9080558

**Published:** 2019-08-15

**Authors:** Lauren E. Edwards, Kate J. Plush, Cameron R. Ralph, Rebecca S. Morrison, Rutu Y. Acharya, Rebecca E. Doyle

**Affiliations:** 1 Animal Welfare Science Centre, Faculty of Veterinary and Agricultural Sciences, University of Melbourne, Parkville, VIC 3052, Australia; 2 SunPork Solutions, Shea-Oak Log, SA 5371, Australia; 3 South Australian Research and Development Institute, Roseworthy, SA 5371, Australia; 4 Rivalea Australia, Corowa, NSW 2646, Australia

**Keywords:** Alfalfa, anticipatory behaviour, farrowing, lactation, nesting behaviour, welfare

## Abstract

**Simple Summary:**

This study investigated how access to a high-value enrichment item (lucerne hay) impacted the welfare of sows in farrowing crates during nesting, farrowing and lactation. Compared to the control group (no nest building material), in which sows in farrowing crates were given 1 kg of lucerne hay daily, they performed more nest-building and less stereotypical behaviour in the 18 h prior to farrowing and gave birth to fewer stillborn piglets. Shortly after farrowing, the control sows performed less interactions with their piglets during early lactation than they did during late lactation, suggesting a reduction in early maternal behaviour for this group. The lucerne sows continued to interact with the lucerne during the 17-day lactation period, indicating that they still found this enrichment rewarding even after the nest-building period had finished. Based on these results, lucerne enrichment was considered to improve sow welfare during farrowing and throughout lactation. These results support the provision of lucerne enrichments under confined farrowing conditions that continue through lactation to improve both sow welfare and piglet survival.

**Abstract:**

This study investigated the effects of providing lucerne hay on the behaviour and the performance of sows housed in farrowing crates during farrowing and lactation. Seventy-two mixed parity sows received either 1 kg lucerne hay daily from entry into the farrowing crate (−2 d from expected farrowing date) until weaning at 17 d (lucerne group, n = 36), or received no additional enrichment (control group, n = 36). In the 18 h prior to farrowing, the sows in the lucerne treatment spent more time performing nest-building behaviour (14.8% lucerne vs 11.1% control, *p* = 0.0009) and less time sham-chewing (1.0% lucerne vs 1.9% control, *p* = 0.01) than control sows, and gave birth to fewer stillborn piglets/litter (0.1 lucerne vs 0.4 control, *p* = 0.027). After farrowing (Day 3), the control sows spent less time lying than the lucerne sows (26% control vs 43% lucerne, *p* < 0.05). The control sows also spent less time interacting with their piglets during early lactation compared to late lactation (25.5% Day 5 vs 47.3% Day 12, *p* < 0.05), suggesting reduced maternal behaviour in this group. The lucerne sows continued to interact with the lucerne throughout lactation, indicating that they still found the enrichment rewarding after the nesting period had ceased. Based on these results, lucerne enrichment was considered to improve sow welfare during farrowing and lactation and reduce the number of stillborn piglets.

## 1. Introduction

Sows are highly motivated to build a nest prior to farrowing, and there has been much research devoted to the benefits and optimal delivery methods of providing nesting materials to pre-parturient sows. While the motivation to build a nest ceases after parturition, sows may continue to need environmental enrichment during lactation to satisfy other motivations, such as foraging and exploration [1,2]. This is particularly relevant for sows that are confined to a farrowing crate during the lactation period due to the lack of enrichment in the farrowing crate environment, as well as the potential for the metabolic cost of feeding a growing litter to stimulate hunger and the motivation to forage [3,4,5,6].

Several studies have investigated the use of straw as an enrichment during lactation but have reported mixed findings. Providing straw for 29–35 days during lactation did not alter the stress physiology or immune function in sows [7,8] but was associated with a reduction in sham chewing [8,9]. Straw also increased the performance of foraging and maternal behaviour shown by the sows [9,10], but produced few other differences in sow behaviour during lactation. In fact, sows have shown little interest in wooden or straw enrichments during the first three weeks of lactation [3,9], and enrichment use reported in late lactation has been attributed to increasing hunger levels associated with the high metabolic demands of milk production [3,11]. While providing enrichments during lactation can have benefits for piglets in terms of improving their response to weaning and reducing the incidence of tail-biting [1,12,13], the benefits for sows are less clear cut.

A possible reason for the lack of clear benefits associated with access to straw enrichment during lactation is the low nutritional value of straw. All the studies described above used straw or wood as enrichments, which are manipulable but do not provide additional nutrition. A manipulable enrichment that provides nutritional benefits may not only satisfy the nesting and foraging motivations of sows, but may also reduce their hunger during late gestation, when they are restrictively fed [4,14], and during late lactation, when they are providing sustenance for a growing litter [15]. Providing a nutritional enrichment may also stimulate additional feed intake by the sow, as varying the sensory properties of the diet can increase the feed intake of suckling pigs [16] and humans [17]. Lucerne hay is a valuable fodder item for livestock due to its high protein and nutrient content [18], and may provide a high-value enrichment item for sows during the confinement period surrounding farrowing and lactation.

Many of the above-mentioned studies assessed sow welfare using traditional measures (time budgets and production data) but few have incorporated cognitive assessment of affective state. Animals can anticipate future events and can be trained to recognise cues that signal the arrival of a pleasant or aversive experience. The anticipation of a positive or negative event results in behavioural change that can be measured; animals show withdrawal and reduced activity when anticipating an aversive experience, but show increased activity and investigative behaviour when anticipating a pleasant experience [19]. The value of the anticipated resource is dependent on the internal state of the animal, and thus, the amount of anticipatory behaviour shown has been proposed as an indicator of positive affect in animals [19,20]. This means that positive affect can be indicated by increased activity in animals that are anticipating a reward, such as a feeding event.

The aim of this study was to investigate the effects of providing lucerne hay as a high value enrichment on the time budgets, anticipatory behaviour and production of confined sows during the farrowing and lactation periods. It was hypothesised that providing enrichment in the form of lucerne hay would have positive impacts on sow welfare at farrowing that would continue throughout lactation and that this would then benefit piglet survival.

## 2. Materials and Methods

This experiment was approved by the Primary Industries and Regions South Australia (PIRSA) Animal Ethics Committee (Project Number: 08–15). The preliminary results of this study have been published elsewhere [21].

### 2.1. Subjects, Housing and Treatments

The experiment was conducted from March 2016 until August 2016 at the Roseworthy Piggery, South Australia. Seventy-two Large White x Landrace mixed parity sows (Mean parity 0.6 ± 0.08 SEM, range P 0 = 35, P 1 = 28, P 2 = 8, unknown = 1) farrowed over six replicates (12 sows per replicate).

The farrowing shed was maintained at an optimal internal temperature using a centrally controlled heating and ventilation system and an evaporative cooling system. The rooms were lit with standard fluorescent lights, with a lighting schedule of 10 h light, 14 h dark. The farrowing shed held five separate rooms, each containing 12 standard farrowing crates. The farrowing crate was 1.8 × 2.4 m in size, contained a sow stall of 0.7 × 2.4 m, a sow feeder and water nipple, plastic slatted flooring and a piglet creep area with solid pad heating. The sows were housed in the same room over all six replicates.

The sows were moved into the farrowing shed at 108 ± 2 days of gestation. On movement into the farrowing crates, the sows were allocated to either a control or lucerne treatment to ensure equal parity distribution and treatment distribution within the room. The sows in the lucerne treatment received 1 kg of lucerne daily leading up to farrowing after feed delivery, and every second day following farrowing. The lucerne was provided on the floor directly in front of the feeder. The control treatment received standard husbandry with no access to nesting materials or enrichment. The sows were fed a lactation pellet (13.8 MJ DE/kg) at 3 kg/day until the day of farrowing, after which they received 5 kg feed in the morning and a maximum of 5 kg in the afternoon (adjusted to appetite). Piglet procedures (fostering, iron injection, oral coccidiostat and tail docking) were conducted by farm staff at 12–24 h of age and weaning occurred at 17.2 ± 0.4 days (mean ± SEM) of age.

### 2.2. Sow Behaviour Measurements

Each farrowing crate had a permanently installed single overhead camera (3MP fixed lens IP dome camera) connected to the 16 channel NVR with infrared capabilities to allow visual recording at night. Sow behaviour was recorded continuously and the following footage was kept for analysis: 24 h prior to farrowing (nesting behaviour), during farrowing (farrowing duration and piglet intervals), after the delivery of lucerne (enrichment use) on −2, +5 and +13 days post-farrowing, and during feeding (anticipatory behaviour) on −2, +3 and +12 days post-farrowing. Five sows were excluded from the nesting and farrowing observations: three of these sows were not pregnant, and two had missing video footage. These sows were included in the observations for enrichment use and anticipatory behaviour. The data from replicate 1 was excluded from the analyses for the anticipatory test due to missing data. The video footage was analysed by two people: observer 1 analysed the nesting and farrowing footage using Windows media player and the enrichment use footage using The Observer XT software (Version 11, Noldus, Wageningen, The Netherlands). Observer 2 analysed the anticipatory behaviour footage, using the same Noldus Observer software. Due to the visibility of the lucerne hay in the farrowing crates, these observers could not be blinded to the treatments.

### 2.3. Nesting Behaviour

The nesting behaviour of the sows (n = 67) was analysed for 18 h prior to the birth of the first piglet, using 15-min instantaneous samples (73 observations per sow). Five sows were only observed for a reduced period (18–71 observations) due to poor visibility or missing video files. Sow behaviour was recorded using the ethogram presented in Table 1. The behaviours indicative of pain were provided by Ison et al. [22]

### 2.4. Farrowing Behaviour

The farrowing behaviour of each sow (n = 67) was observed continuously from the birth of the first piglet until the placenta was passed, or if the placenta could not be observed, then observations ceased at the time the last piglet was born. These observations allowed the farrowing duration to be calculated (both first piglet to placenta, and first piglet to last piglet), as well as the average piglet interval. The number of stillborn piglets was also recorded, with a stillborn piglet classified as a piglet that never moved after being born.

### 2.5. Enrichment Use

Sow behaviour following the delivery of lucerne was recorded for 20 min following delivery on days −2, +5 and +13 days post-farrowing for a subset of sows (n = 12 lucerne and n = 12 control). Sow behaviour during this 20 min period was analysed using the ethogram presented in Table 2, using 15 s instantaneous samples (a total of 80 observations per sow). The latency to interact with the enrichment and the duration of enrichment use was also recorded. The period of enrichment use was considered to have ceased when the sow had not touched the enrichment for at least 5 min.

### 2.6. Anticipatory Behaviour

On Days −2, +3 and +12 post-farrowing, the anticipatory behaviour of all sows in replicates 2–6 (n = 60) was tested by walking the feed cart past the sows without feeding them. Once the feed cart had passed all of the pens the stockperson waited for 3 min before commencing the normal feed delivery. The behaviour of the sows during this anticipatory period was recorded.

The behaviour of the sows was recorded continuously using the ethogram presented in Table 3, starting 2 min prior to the stock people entering the shed and finishing when the focal sow received feed. This meant that each sow was observed for a different duration, as sows fed first would have a shorter observation period than sows fed last. The 2-min period prior to the stock people entering the shed was called the Pre-Test period and was used as a baseline measure of sow behaviour. The period between the stock people entering the shed and the sow receiving feed was called the Test period and was used to measure the anticipatory behaviour of the sows to feed delivery.

### 2.7. Production Measurements

Production data were collected for the number of piglets born (total, number born alive, number born dead), the number of piglet deaths, and the number of piglets weaned per sow. Subsequent reproduction data for each sow was collected for weaning to oestrus interval (days), mated next batch (%), pregnancy rate (%), farrowing rate (%), and total piglets born, piglets born alive, and piglets born dead in the subsequent litter. Piglet survival was calculated on a per sow basis and included fostered piglets.

### 2.8. Statistical Analysis

#### 2.8.1. Anticipation, Enrichment Use and Nesting

Statistical analyses were performed using R (R Foundation for Statistical Computing, Vienna, Austria) [23]. Generalised linear models with a quasibinomial distribution (logit link function) were used to analyse the results presented as a proportion (e.g., % nesting behaviour) [24]. Poisson distributions (log link function) were used for count data (e.g., number of behavioural transitions). Linear models were used for the few continuous measures (e.g., total use).

When there were repeated measures, individual sow ID was fit as a random factor and a generalised linear mixed model was used. Examples of this include enrichment use over time and anticipatory behaviour over time.

For all the models, interactions of treatment, parity and replicate were included, as well as the time when it was repeated, to investigate all the relationships. Step-wise reductions of the models were performed to reach the final models.

When analysing simple binary outcomes (enrichment use vs. no use), chi square tests were used. Spearman’s correlations were performed to investigate the correspondence between count-count and count-continuous variables.

#### 2.8.2. Production Analyses

Statistical analyses were performed using a general linear mixed model, using each sow/litter as the experimental unit with parity, treatment and their interaction as fixed effects, and replicate as a random term. This model was applied to the following variables: farrow duration (log10 transformed), piglet interval (log10 transformed), total piglets born, piglets born alive, piglets weaned and weaning to oestrus interval. A covariate of total number of piglets born was added to the model for farrowing duration and piglet interval only. The same model was used for the number of piglets born dead and post-natal piglet deaths but a generalised linear model with Poisson distribution was applied, and a binomial distribution was applied to the number of sows that were mated in the next batch, pregnancy rate and farrowing rate.

## 3. Results

### 3.1. Nesting Behaviour

Of the 35 focal sows that received the lucerne treatment, 30 interacted with it during the observed nesting period (85%). For the sows that interacted with the lucerne, the median number of interactions was 4 (out of 73 observations), the maximum recorded was 12, and the mean was 4.3, representing 6% of the sows’ total time budget.

The treatment effects on nesting behaviour are presented in Table 4. When the vacuum nesting behaviour of the sows was compared between the two treatments, there was a tendency for sows in the Control group to spend more time performing vacuum-nesting behaviours than the enriched sows (*p* = 0.08). When enrichment use was combined with vacuum nesting behaviours, there was a significant effect of treatment on the total time spent nesting (*p* = 0.009) with sows with lucerne spending significantly more time performing nesting behaviours than control sows. The final treatment effect was that control sows spent significantly more time sham chewing than sows in the lucerne treatment (*p* = 0.01). No other treatment-related differences were identified.

The parity effects on nesting behaviour are presented in Table 5. Parity 1 sows were significantly more active prior to farrowing than Parity 2 sows (*p* = 0.021) and showed significantly more sham chewing (*p* < 0.001) and bar biting (*p* = 0.002) than Parity 0 sows. Parity 2 sows also showed more bar biting than Parity 0 sows (*p* = 0.002).

The incidence of pain-related behaviours was low (average 2.3%) and was not affected by treatment or parity. Farrowing duration and the number of stillborn piglets were not significantly (*p* > 0.05) correlated with the performance of nesting behaviours or the amount of activity shown during the nesting period.

### 3.2. Enrichment Use

In terms of main effects, the control sows spent significantly more time inactive following lucerne delivery than the lucerne sows (Table 6, *p* < 0.001). There were no other main effects of treatment. The sows that received the lucerne treatment showed significant effects of test day on the duration of lucerne use (*p* = 0.050; Table 7), and the number of times they interacted with the lucerne (*p* = 0.02). These sows interacted with the lucerne in fewer bouts but for longer durations during the late lactation period (Day +13). All the sows were more active following lucerne delivery during the late lactation period (Day +13, *p* < 0.001) and showed the highest amount of oral manipulation during the pre-farrowing period (Day −2, *p* < 0.001).

Significant test day x treatment interactions occurred for the amount of sow activity, feeding and interactions with the piglets that occurred following lucerne delivery (Table 8). Sow activity was represented by the composite variable ‘Active behaviour’, which was created by summing the active behaviours that were not involved in enrichment use: Pawing, Rooting/Nosing, Active with Piglets, Sniff Floor and Oral Manipulation. During early lactation (Day +5), sows in the lucerne treatment performed significantly less active (non-enrichment) behaviours compared to late lactation (Day +13, *p* = 0.0002), while the control sows performed significantly less total piglet interactions (active and passive) in early lactation (Day +5) compared to late lactation (Day +13, *p* < 0.001). All the sows showed an increase in feeding behaviour following lucerne delivery in late lactation (Day +13), but the control sows spent more time feeding than the lucerne treatment (*p* = 0.003).

### 3.3. Anticipatory Behaviour

The total number of behaviours that each sow displayed during each phase of the anticipatory test was divided by the number of minutes for the pre-test and test phases to create the summary variable ‘Behavioural transitions/min’. The sows showed more behavioural transitions (test × day interaction *p* = 0.052; Table 9) and spent proportionally less time lying (test × day interaction *p* < 0.001) during the anticipatory test period compared to the pre-test period, indicating that the procedure had successfully elicited anticipatory behaviour.

There was no influence of treatment on anticipatory behaviour. The number of behavioural transitions per minute changed over days (*p* < 0.001; Table 10), with it significantly (*p* < 0.05) declining each day from the pre-farrowing period (Day −2, mean transitions/min = 10.6 ± 0.57 SEM) to early lactation (Day +3, mean transitions/min = 9.0 ± 0.57 SEM) to late lactation (Day +12, mean transitions/min = 6.7 ± 0.57). The proportion of time spent lying was significantly influenced by treatment × day (*p* < 0.001; Table 10). The amount of time spent lying increased as the experiment progressed for both treatments, with the sows in the lucerne treatment spending more time lying than the control sows during early lactation (Day +3).

### 3.4. Farrowing Behaviour and Reproductive Performance

The sow farrowing behaviour and production data are presented in Table 11. The number of piglets born dead per litter was reduced by 0.3 piglets in the lucerne treatment (*p* = 0.027). There were no other significant treatment differences for any of the reproductive measures recorded during this experiment.

A significant parity × treatment interaction occurred for the weaning to oestrus interval (*p* = 0.034). Sows displaying oestrus within one week of weaning were bred, whilst the remainder were not bred that batch. More gilts from the lucerne treatment were mated immediately following weaning (81%) than controls (60%), but this relationship was reversed in multiparous sows (lucerne 67% vs control 90%).

## 4. Discussion

The hypothesis was accepted in the current experiment, as providing sows in farrowing crates with lucerne hay prior to farrowing and throughout lactation was associated with several measures of positive sow welfare. The findings of reduced stereotypies, fewer stillborn piglets and increased maternal behaviour indicate that lucerne can improve both sow welfare and piglet survival, particularly during the nesting and farrowing period.

Providing lucerne significantly reduced the total number of stillbirths by 0.3 piglets per litter. This could equate to an additional 30 piglets born alive for every 100 sows farrowed, and there are several possible explanations for this result. The first is that the provision of nesting materials may have reduced stress in the lucerne treatment sows, leading to a higher concentration of circulating oxytocin in this group [25,26]. As oxytocin stimulates uterine contractions, this may have facilitated better farrowing outcomes for the piglets [25]. In the current experiment, there was mixed evidence that the reduced rate of stillbirth was due to a reduction in circulating oxytocin, based on behavioural indicators of oxytocin only. There were no treatment differences in the piglet interval or farrowing duration, however maternal behaviour did appear to decrease in the control treatment during early lactation.

An alternative explanation for the reduction in stillbirth may relate to the nutritional benefits of lucerne hay for the sows and their piglets. The additional nutrition may have improved the viability of the piglets by increasing their birth weight [27], although piglet weights were measured post-fostering and thus, treatment effects could not be tested here. Feeding sows a high fibre diet for two weeks prior to farrowing has been shown to reduce the rate of stillborn piglets [28], and this effect may be due to a reduction in constipation for the sows, as the hard feces can act as a physical barrier in the birth canal [29,30]. Furthermore, the additional energy provided by the fermentation of fibre in the hindgut may provide a more stabilized and prolonged source of energy for the sows than that provided by concentrated feed alone [28]. As farrowing is a strenuous process, the farrowing duration and stillbirth rate have been shown to linearly increase with the time since last meal [31]. It is possible that the additional fibre and energy provided by the lucerne enrichment was, while small, sufficient to provide a benefit for piglet survival. This is supported by a weak but significant correlation between the amount of stereotypic behaviour (sham chewing) displayed by the sows in the current study and their rate of stillbirth (r = 0.3, *p* = 0.02), similarly to that reported by Von Borrell and Hurnik [32]. It is also possible that maternal stress, as indicated by stereotypical behaviour, played a role in the unexplained treatment effect on the rate of stillborn piglets in the current study but was not captured by the variables measured. Future research using lucerne hay as an enrichment for confined sows could monitor piglet birth weights, sow energy status and sow stress physiology to determine whether the observed treatment effect on the rate of stillborn piglets is due to a nutritional benefit or an endocrine dysfunction of the sow.

When lucerne was available, 85% of sows chose to interact with it, and compared to control sows, lucerne-enriched sows performed more total nesting behaviours. This result agrees with the literature, which suggests that once the sow is hormonally motivated to begin performing nesting behaviour, the availability of appropriate nesting material is influential in releasing the entire nesting repertoire [33,34]. It is unknown why 15% of sows were not observed to interact with the lucerne during the nesting period. Potentially, these sows may simply not have found the lucerne to be an attractive nesting substrate. Alternately, sows that interacted with the enrichment only infrequently may have been missed due to the 15-min sampling schedule, or the sows may have consumed the lucerne so rapidly that it was gone before the second observation took place (15 min after delivery).

The performance of nesting behaviours has been linked to positive welfare outcomes for confined sows, including reduced injuries, stress and stereotypical behaviours such as bar biting [35]. As discussed above, nesting behaviour stimulates oxytocin release, which, in turn, promotes maternal behaviour following parturition [36,37]. While oxytocin concentrations were not measured in this study, sows in the control group did interact with piglets less during early lactation (day +5 enrichment use time budget). In addition, the control sows showed more active behaviours and less lying during early lactation, based on the enrichment use time budgets (day +5) and the anticipatory behaviour tests (day +3). Post-parturient sows typically show an increase in lying behaviour in the two days following parturition [38], and the increased activity shown by the control sows during early lactation combined with fewer piglet interactions may indicate a dysfunction in maternal behaviour. These behaviours are consistent with the reduced maternal behaviour seen in sows that are deprived of nesting materials prior to parturition [36,39]. While piglet outcomes were not assessed as part of this study, increased maternal interactions and lying behaviour are likely to be beneficial for piglet survival and growth, and the provision of lucerne enrichment may be beneficial for both sows and piglets in confinement housing.

In terms of stereotypical behaviour, sham chewing during the nesting period was performed at a higher rate in the control treatment than the lucerne treatment, while bar-biting was not affected by treatment. This effect appears to be specific to the nesting period, as there were no treatment differences in the rate of oral manipulation of the pen fittings (including sham chewing) prior to the nesting period (Day −2 enrichment use time budget) or throughout lactation. Sham-chewing in pigs appears to be a redirected foraging activity that is motivated by hunger [4,40], although there is evidence that this behaviour can vary according to individual differences in the response of pigs to suboptimal housing [41,42]. The lack of treatment effects outside the nesting period suggests that the reduction in sham chewing during the nesting period may have been associated with a reduction in nesting specific motivations or discomfort, rather than hunger. This effect was most apparent for P 1 (n = 28) sows, who displayed the highest level of activity and performed much higher rates of sham chewing during the nesting period than the P 0 (n = 35) and P 2 sows (n = 8). Both P 1 and P 2 sows also showed higher rates of bar biting than the P 0 sows. While the sample size for the P 2 sows was much smaller than that of the P 0 and P 1 sows, the differences reported here relate to a reduction in stereotypies shown by the P 0 sows rather than differences occurring in the smaller P 2 group. The differences occurring between the P 0 and P 1 sows, which have similar group sizes, indicate that this is a real effect rather than a statistical anomaly. This results are consistent with previous research in which gilts showed less stereotypies than older sows during nesting [43] and may be caused by the novelty of farrowing and the crate confinement interfering with nest-building motivation and behaviour in gilts. It also supports the view that stereotypies may represent periparturient discomfort, as multiparous sows may be experiencing a greater motivation for nest building due to increased prolactin concentrations [36], and possibly increased hunger [44,45], in comparison to gilts. Little information has been published on the relationship between sham chewing and nesting in sows, therefore, further interpretation of this effect is limited. However, based on the reduction of stereotypical behaviour observed in the treatment group, the provision of lucerne appears to enhance the welfare of sows during the nest-building period.

The provision of lucerne hay as an enrichment had clear benefits for sows during nesting and farrowing, however the benefits for sows during the 17-d lactation period were less clear. Sow welfare during lactation was assessed using the 20-min time budgets of the sows following lucerne delivery, and the sows’ anticipatory behaviour toward feed. Following lucerne delivery, the greater activity levels and behavioural repertoire displayed by sows in the lucerne treatment likely reflects the increased complexity of their lucerne-enriched environment. All the sows showed an increase in activity over the course of lactation, which coincides with the period that the sow would leave the nest and return to the herd under natural conditions [46] and may be associated with an increased motivation to forage and explore [3,9,47]. It should be noted that these studies used much longer lactation periods than the current study. It appears that the sows in the treatment and control groups in the current study expressed their motivations to forage and explore differently, although caution must be used when interpreting the results from the relatively brief observation period (20 min) and small sample size (n = 24). During late lactation, the sows in the lucerne treatment group were more active and displayed longer bouts of lucerne use than the sows in the control group, which is consistent with using the lucerne as a feeding or foraging substrate. The control sows spent more time feeding, possibly stimulated by the sounds and smells of adjacent sows consuming lucerne, or alternately, using the feed as a foraging substrate. Whether foraging in lucerne hay represents a better welfare outcome than foraging in feed is yet to be determined, but sows in the lucerne treatment were choosing to forage in the lucerne hay when both lucerne and feed were available.

In addition to the treatment effects described above, the sows that received the lucerne treatment altered their pattern of enrichment use over time; these sows interacted with the lucerne in many short bouts during early lactation, and for fewer, longer bouts, during late lactation (average bout length = 63 s pre-farrowing, 47 s early lactation, 93 s late lactation, calculated from the values in Table 7). This suggests that the lucerne became more rewarding to interact with during late lactation. While this may represent increased motivation to forage due to hunger, it should be noted that during the same period, the sows showed less anticipation of feed, which suggests that they were less hungry. The duration of performing a pleasurable activity has been associated with an affective state in mice, with longer durations being associated with positive affective states [48]. If bout duration reflects reward value and affect, this could indicate an improvement in the affective state of sows in the lucerne treatment during late lactation.

The current study is one of the first to attempt to detect changes in affect due to housing conditions on sows. Sows in both treatments showed similar levels of anticipation toward feed delivery, suggesting that the lucerne enrichment was not sufficiently different to the control treatment to generate a change in affective state prior to feeding. A comparison between this result and the literature is difficult, as most studies of anticipation in pigs have examined young pigs while anticipating a positive or negative event, which the pigs have been trained to anticipate, rather than housing conditions [49,50,51,52,53]. Mahnhardt et al., [54] assessed the anticipatory response of pigs to their daily feed routine and found reduced lying in the 30 min prior to feeding for pigs, similarly to the reduction in lying seen in the current study. This, combined with the increased activity seen in the sows during the testing phase, confirms that using gross measures of activity can detect anticipatory behaviour in pigs; however, further research is needed to explore the effects of housing conditions and enrichment on the anticipatory response.

Despite the lack of treatment effects, the anticipatory behaviour of sows declined over time and the decreased anticipation of feed suggests that feed became less rewarding during the course of the experiment. This is likely due to the increased feed allowance that the sows received during lactation (3 kg/day during gestation and 5–10 kg/day during lactation) and sows spent more time feeding in late lactation (based on the time budgets for enrichment use). Evidently, the use of anticipatory behaviour as a measure of sow welfare requires further refinement before it can be used as a definitive indicator of sow affective state under commercial conditions. Fasting the sows prior to feed delivery during late lactation (when feed was available *ad libitum*) may have provided a more accurate measure of anticipatory behaviour than was used in the current study.

The subsequent reproductive performance of sows was influenced by treatment, but this was not consistent across parities. Primiparous sows typically have a longer weaning-to-oestrus interval (WOI) and a lower mating success rate post-weaning than multiparous sows [55], and this was reflected by the number of sows that mated immediately (<7 days) after weaning in the control treatment (67% of primiparous sows (n = 12) vs 90% of multiparous sows (n = 19)). This relationship was reversed for sows in the lucerne treatment, with primiparous sows (n = 16) displaying an increased rate of successful mating (81%) and the multiparous sows (n = 13) showing a decreased rate (60%), relative to the controls. Feeding legume fibre (lupins) prior to mating is beneficial for oocyte maturation in primiparous sows [56], and can explain the improvement in mating success rate for these animals. The 30% decrease in mating success seen in the multiparous sows given lucerne is consistent with a suboptimal lactation diet, possibly related to the dilutive effects of fibre on nutritional intake [57,58], but it is not clear why this effect would not also apply to the primiparous sows that are under more severe metabolic challenges during lactation. The same treatment x parity interaction for the WOI was reported by Poulopoulou, et al. [59] when providing sows with the same daily feed allocation in two or three meals per day, but the authors could not explain this effect conclusively.

## 5. Conclusions

Lucerne enrichment improved sow welfare during farrowing and lactation and improved piglet survival. Sows provided lucerne exhibited more maternal behaviours (more nesting and interactions with piglets, greater behavioural repertoire), reduced indicators of frustration and discomfort (sham chewing) and preferred to forage in lucerne rather than feed. Sows fed lucerne also had less stillborn piglets. The relationship between sow anticipatory behaviour toward feed and sow affective state was unclear and needs further research. Furthermore, the nutritional value of enrichment requires further investigation. In conclusion, enrichments that continue through farrowing and lactation should be adopted in farrowing systems that utilise crates to improve both sow welfare and piglet survival.

## Figures and Tables

**Table 1:** Ethogram of the behaviours used to record nesting behaviour in sows during the 18 h prior to farrowing.

Behaviours	Definition
Lateral lying	Laying laterally with shoulder resting on ground
Sternal lying	Laying with sternum on ground
Sit	Sitting on rump, forelegs straight
Kneel	Kneeling on forelegs, standing with hind legs. Generally occurred as sow was lying down
Stand	Standing still on all four feet
Stepping	Taking one or more steps
Inactive	No behaviour of interest is being performed (idle, watching stockperson, etc.)
Eat	Head in feeder with feed present. Look for rhythmic chewing motions (cheeks or ears) and standing still at the feeder. Fed in early morning.
Drink	Mouth in contact with drinker. There are two drinkers (high and low) - sow may drink from any posture. Look for standing still with the mouth angled up and in contact with the drinker, with rhythmic swallowing movements of cheeks and ears. You may see some water trickling down near the mouth
Eliminate	Urination or defecation. Sow may show a small squatting/rounded rump position while urinating
Sniffing floor	Moving snout over floor where no lucerne is present, just before lying down. Little movement of head
Feeder interaction	Bites or rubs head against feeder, pushes or roots against feeder, head in empty feeder. No standing and chewing
Drinker interaction	Chewing on drinker or pushing snout against drinker to let water out. Obviously not swallowing water (no rhythmic swallowing movements)
Bar interaction	Bites, paws or rubs head against bars, pushes against bars with snout
Nesting behaviour	No lucerne/straw under snout. Sniffing floor, rooting floor, pawing any object - all without touching the enrichment object
Vacuum nesting behaviour	A composite variable, created by summing all nesting-related behaviours that don’t use enrichment (feeder/drinker/bar interactions, and nesting behaviour)
Enrichment interaction	Must be touching the enrichment object. Enrichment can be in the feed trough or on the floor. Sniffing, chewing, pawing at, carrying in mouth, rooting
Other behaviour/ Sham chewing	Sham chewing (rhythmic movement of head or ears with no feed or enrichment visible in the mouth, and no prior feeding or enrichment interaction), interacting with stockperson etc.
Strain	Body clenches, with hind quarters drawing toward belly and/or back tensing
Pain indicators	Tail flick, leg lift (one or both hind legs lift toward belly), shaking, front leg row (rowing motions)

**Table 2:** Ethogram used to record the time budgets of all sows for 20 min following lucerne delivery on Days −2, +5 and +13.

Behaviour	Definition
Stand	Standing still on all four feet
Kneel	Kneeling on forelegs, standing with hind legs. Generally occurred as sow was lying down
Sit	Sitting on rump, forelegs straight
Lie	Lying laterally or sternally
Step	Walking more than two steps forward or backward
Chewing enrichment	The mouth is visibly opening and closing while chewing, and/or has lucerne sticking out of the mouth, or snout and ears are moving rhythmically with snout above lucerne (when mouth is not visible)
Sniff enrichment	Sow is moving snout over lucerne. She may be eating/chewing it, but mouth is not visible. No upward thrusting movements like rooting
Rooting enrichment	Sow is making upward thrusting movements with her snout while her snout is in the lucerne, lifting the lucerne up off the ground
Pawing at enrichment	Sow is moving one foreleg forward and backward to move the lucerne. This may occur while standing or lying
Drinking	Sow had mouth or snout in the position of the drinker and remained motionless except for small regular movements of the jaw or ears that indicated swallowing
Feeding	Sow had head in feed trough in the presence of food
Inactive	Sow was not performing any of the other behaviours on this ethogram. Includes urination/defecation and watching stock people in the shed.
Pawing	Sow is moving one foreleg back and forth, dragging the foot across a surface, with no lucerne present. This may be the floor or the feed trough
Rooting/nosing	The sow is pushing her snout around on the floor or another surface. There may be forward thrusting movements as for rooting. There is no lucerne present
Oral manipulation	The sow is manipulating an object with her mouth that is not feed, the drinker or lucerne. This includes bar biting and placing her head in the empty feed trough
Sniff floor	Sow points snout directly down toward the floor. It may be still or moving. There is no lucerne on the part of the floor that she is sniffing
Active with piglets	Sow contacts a piglet with her snout or extends her snout toward a piglet that is out of reach. May include both positive and negative interactions
Suckling	Sow is lying on her side and >90% of her litter are suckling. If less than 90% are suckling, then it is classified as a passive interaction with the piglets (see below)
No piglet interaction	None of the piglets are touching the sow with their snout
Passive with piglets	At least one piglet is touching the sow anywhere on her body with their snout. The piglet must be awake - piglets sleeping in contact with the sow were not included

**Table 3:** Ethogram of the behaviours recorded continuously during the anticipatory test conducted on Days −2, +3 and +12**.**

Behaviour	Definition
Lateral laying	Laying laterally with shoulder resting on ground
Sternal laying	Laying with sternum on ground
Sitting	Sitting on rump, forelegs straight
Standing	Standing still on all four feet
Head left corner	Snout is in the far left corner of the crate, above the feed trough
Head right corner	Snout is in the far right corner of the crate, above the feed trough
Head between bars	Snout between side rails. Includes when lying down or suckling
Head in feeder	Snout in feeder, past line of front of crate
Head above bars	Head above level of top rail
Drinking	Snout on nipple drinker
Scratch side	Rub side up and down against side bars of crate
Bar biting	Mouth open and around bar anywhere on crate, or pushing snout against bars
Suckling	Lying lateral with all piglets at teats (also scored when up to 2 piglets not at teats)

**Table 4:** Treatment differences in proportions of nesting behaviours (% mean) displayed for 18 h prior to farrowing (transformed mean ± SEM)**.**

Behaviour	Lucerne (n = 35)	Control (n = 32)	*p*-Value
	Mean %	Mean %	
Vacuum nesting behaviours only	9.0	11.1	0.08
Vacuum nesting behaviours + lucerne use	14.8 (−1.75 ± 0.08)	11.1 (−2.08 ± 0.10)	0.0009
Lateral lying	52.0	53.0	0.67
Inactive	80.7	83.1	0.11
Pain behaviours	2.4	2.1	0.69
Bar biting	2.19	2.61	0.28
Sham chewing	1.0 (−0.95 ± 0.38)	1.9 (−0.69 ± 0.37)	0.01

**Table 5:** Parity differences in proportions of nesting behaviours (% mean) displayed for 18 h prior to farrowing (transformed mean ± SEM)**.**

Behaviour	Parity 0 (n = 35)	Parity 1 (n = 28)	Parity 2 (n = 8)	*p*-Value
V	9.0	11.2	8.0	0.39
Nesting behaviours + lucerne use	13.6	13.2	10.1	0.30
Lateral lying	53.4	50.3	54.6	0.52
Inactive	82.5 ^ab^ (1.55 ± 0.1)	79.7 ^a^ (1.37 ± 0.1)	86.5 ^b^ (1.86 ± 0.2)	0.02
Pain behaviours	2.5	2.0	1.9	0.69
Bar biting	1.3 ^a^ (−0.06 ± 0.2)	3.4 ^b^ (0.84 ± 0.1)	3.9 ^b^ (1.01 ± 0.2)	0.002
Sham chewing	0.4 ^a^ (−0.14 ± 0.3)	3.1 ^b^ (0.77 ± 0.1)	0.2 ^a^ (−2.09 ± 1.0)	*p* < 0.001

^ab^*p* < 0.05.

**Table 6:** Treatment differences in behavioural time budgets following the provision of lucerne (transformed mean ± SEM)**.**

Behaviour	Lucerne (n = 12)	Control (n = 12)	*p*-Value
Oral manipulation (count)	2.3	2.6	0.45
Inactive	39.9 (3.7 ± 0.05)	63.8 (4.1 ± 0.05)	<0.001

**Table 7:** Test day differences in behavioural time budgets of all sows (n = 24) following the provision of lucerne (Transformed mean ± SEM). The results for the frequency and duration of lucerne use only apply to the treatment sows.

Behaviour	Pre-Farrowing Day −2	Early Lactation Day +5	Late Lactation Day +13	*p*-Value
Frequency of lucerne use (count)	9.8 ^a^ (2.2 ± 0.16)	12.8 ^b^ (2.4 ± 0.16)	9.2 ^a^ (2.1 ± 0.16)	0.02
Duration of lucerne use (s)	617 ± 103.0 ^a^	605 ± 103.0 ^a^	859 ± 103.0 ^b^	0.05
Oral manipulation (count)	4.5 ^a^ (0.5 ± 0.4)	1.2 ^b^ (-0.4 ± 0.4)	1.8 ^b^ (-0.8 ± 0.4)	<0.001
Inactive (%)	56.9 (4 ± 0.04) ^a^	55.0 (4 ± 0.04) ^a^	43.6 (3.7 ± 0.05) ^b^	<0.001

^ab^*p* < 0.05.

**Table 8:** Test day x treatment interactions for all sows following lucerne delivery during pre-farrowing (Day −2), early lactation (Day +5) and late lactation (Day +13) (transformed mean ± SEM).

Behaviour	Test Day	Control (n = 12)	Lucerne (n = 12)
Active behaviour	−2	7.7 (1.9 ± 0.21) ^ab^	5.6 (1.5 ± 0.22) ^ab^
	+5	8.3 (2 ± 0.21) ^ab^	3.9 (1.2 ± 0.23) ^a^
	+13	6.3 (1.7 ± 0.21) ^ab^	8.2 (1.9 ± 0.21) ^b^
Feeding behaviour (count)	−2	1.3 (-0.7 ± 0.) ^ab^	0.25 (-2.7 ± 0.86) ^a^
	+5	0.16 (-2.8 ± 0.92) ^ab^	0.91 (-1.4 ± 0.71) ^a^
	+13	9.25 (1.2 ± 0.60) ^c^	3.4 (-0.1 ± 0.66) ^bc^
Piglet interactions (%)	+5	25.5 ^a^	42.6 ^ab^
	+13	47.3 ^b^	45.8 ^ab^

^acb^*p* < 0.05.

**Table 9:** Test x day interactions for the number of behavioural transitions shown and the proportion of time spent lying by sows prior to feed delivery for the Anticipatory test (transformed mean ± SEM).

Behaviour	Day	Test	
		Pre-Test (n = 60)	Anticipatory Test (n = 60)
Transitions (mean behaviours/min)	−2	4.6 ^a^ (± 0.52)	10.6 ^c^ (± 0.52)
	+3	3.0 ^a^ (± 0.52)	9.0 ^c^ (± 0.52)
	+12	2.7 ^a^ (± 0.52)	6.7 ^b^ (± 0.52)
Proportion lying (mean %)	−2	53% ^a^ (−2.08 ± 0.10)	10% ^d^ (−2.8 ± 0.17)
	+3	79% ^b^ (−2.08 ± 0.10)	28% ^e^ (−1.2 ± 0.17)
	+12	81% ^c^ (−0.69 ± 0.37)	35% ^f^ (−0.68 ± 0.17)

^abcdef^*p* < 0.05.

**Table 10:** Treatment x day interactions for the proportion of time spent lying by sows prior to feed delivery (Transformed mean ± SEM)**.**

Behaviour	Day	Lucerne (n = 30)	Control (n = 30)
Proportion lying (mean %)	−2	10% ^ab^ (−4.84 ± 0.49)	10% ^a^ (−5.27 ± 0.46)
	+3	36% ^e^ (−1.5 ± 0.46)	19% ^bc^ (−2.82 ± 0.42)
	+12	33% ^cd^ (−1.04 ± 0.43)	38% ^de^ (−1.47 ± 0.42)

^abcdef^*p* < 0.05.

**Table 11:** Sow farrowing behaviour and production variables during the experimental period (back transformed mean)**.**

Farrowing Measure	Control (n=31)		Lucerne (n = 33)		*p*-Value
	Mean	SEM	Mean	SEM	
Log mean piglet interval	1.18 (15.1)	0.05	1.24 (17.4)	0.05	0.364
Log total farrowing duration (min)	2.22 (166.0)	0.05	2.31 (204.2)	0.04	0.174
Total piglets born	10.9	0.5	11	0.5	0.834
Piglets born alive	10.4 *	0.5	10.9	0.5	0.45
Piglets born dead	0.4	0.1	0.1	0.1	0.027
Piglet deaths	0.8	0.2	0.6	0.1	0.328
Piglets weaned	10.5 *	0.3	10.6	0.3	0.865
Weaning to oestrus interval (days)	13.2	1.8	11.7	1.9	0.58

*The mean number of piglets weaned is greater than the mean number born alive due to piglet fostering.

## References

[1] Vanheukelom V., Driessen B., Geers R. (2012). The effects of environmental enrichment on the behaviour of suckling piglets and lactating sows: A review. Livest. Sci..

[2] Tuyttens F.A.M. (2005). The importance of straw for pig and cattle welfare: A review. Appl. Anim. Behav. Sci..

[3] Valros A., Pedersen L.J., Pöytäkangas M., Jensen M.B. (2017). Evaluating measures of exploratory behaviour in sows around farrowing and during lactation—a pilot study. Appl. Anim. Behav. Sci..

[4] D’Eath R.B., Jarvis S., Baxter E.M., Houdijk J., Špinka M. (2018). 7 - mitigating hunger in pregnant sows. Advances in Pig Welfare.

[5] Revell D.K., Williams I.H., Mullan B.P., Ranford J.L., Smits R.J. (1998). Body composition at farrowing and nutrition during lactation affect the performance of primiparous sows: Ii. Milk composition, milk yield, and pig growth1. J. Anim. Sci..

[6] Auldist D.E., Morrish L., Eason P., King R.H. (1998). The influence of litter size on milk production of sows. Anim. Sci..

[7] Jarvis S., D’Eath R.B., Robson S.K., Lawrence A.B. (2006). The effect of confinement during lactation on the hypothalamic–pituitary–adrenal axis and behaviour of primiparous sows. Physiol. Behav..

[8] Liu H., Yi R., Bi Y., Li J., Li X., Xu S., Bao J. (2018). Physiology, immunity, stereotyped behavior, and production performance of lactating sows in enriched environment. Int. J. Appl. Res. Vet. Med..

[9] Cronin G.M., Smith J.A. (1992). Effects of accommodation type and straw bedding around parturition and during lactation on the behaviour of primiparous sows and survival and growth of piglets to weaning. Appl. Anim. Behav. Sci..

[10] Bulens A., Renders L., Van Beirendonck S., Van Thielen J., Driessen B. (2014). An exploratory study on the effects of a straw dispenser in farrowing crates. J. Vet. Behav..

[11] Valros A., Rundgren M., Špinka M., Saloniemi H., Rydhmer L., Hultén F., Uvnäs-Moberg K., Tománek M., Krejcí P., Algers B. (2003). Metabolic state of the sow, nursing behaviour and milk production. Livest. Prod. Sci..

[12] Oostindjer M., van den Brand H., Kemp B., Bolhuis J.E. (2011). Effects of environmental enrichment and loose housing of lactating sows on piglet behaviour before and after weaning. Appl. Anim. Behav. Sci..

[13] Yang C.-H., Ko H.-L., Salazar L.C., Llonch L., Manteca X., Camerlink I., Llonch P. (2018). Pre-weaning environmental enrichment increases piglets’ object play behaviour on a large scale commercial pig farm. Appl. Anim. Behav. Sci..

[14] Kim S.W., Weaver A.C., Shen Y.B., Zhao Y. (2013). Improving efficiency of sow productivity: Nutrition and health. J. Anim. Sci. Biotechnol..

[15] Quesnel H., Etienne M., Père M.C. (2007). Influence of litter size on metabolic status and reproductive axis in primiparous sows. J. Anim. Sci..

[16] Middelkoop A., Choudhury R., Gerrits W.J.J., Kemp B., Kleerebezem M., Bolhuis J.E. (2018). Dietary diversity affects feeding behaviour of suckling piglets. Appl. Anim. Behav. Sci..

[17] Rolls B.J., Rowe E.A., Rolls E.T., Kingston B., Megson A., Gunary R. (1981). Variety in a meal enhances food intake in man. Physiol. Behav..

[18] Gaweł E., Grzelak M., Janyszek M. (2017). Lucerne (medicago sativa l.) in the human diet—case reports and short reports. J. Herb. Med..

[19] Spruijt B.M., van den Bos R., Pijlman F.T.A. (2001). A concept of welfare based on reward evaluating mechanisms in the brain: Anticipatory behaviour as an indicator for the state of reward systems. Appl. Anim. Behav. Sci..

[20] Boissy A., Manteuffel G., Jensen M.B., Moe R.O., Spruijt B., Keeling L.J., Winckler C., Forkman B., Dimitrov I., Langbein J. (2007). Assessment of positive emotions in animals to improve their welfare. Physiol. Behav..

[21] Doyle R.E., Ralph C.R., Edwards L.E., Morrison R.S., Cronin G.M., Plush K.J. The reproductive value of enrichment to sows at farrowing. Proceedings of the 16th Biennial Conference of the Australasian Pig Science Association.

[22] Ison S.H., Jarvis S., Rutherford K.M.D. (2016). The identification of potential behavioural indicators of pain in periparturient sows. Res. Vet. Sci..

[23] Team R.C. (2013). R: A language and environment for statistical computing. https://www.gbif.org/tool/81287/r-a-language-and-environment-for-statistical-computing.

[24] McCullagh P. (2019). Generalized Linear Models.

[25] Baxter M.R., Petherick J.C. The effect of restraint on parturition in the sow. Proceedings of the International Pig Veterinary Society.

[26] Lawrence A.B., McLean K.A., Jarvis S., Gilbert C.L., Petherick J.C. (1997). Stress and parturition in the pig. Reprod. Domest. Anim..

[27] Vanderhaeghe C., Dewulf J., de Kruif A., Maes D. (2013). Non-infectious factors associated with stillbirth in pigs: A review. Anim. Reprod. Sci..

[28] Feyera T., Højgaard C.K., Vinther J., Bruun T.S., Theil P.K. (2017). Dietary supplement rich in fiber fed to late gestating sows during transition reduces rate of stillborn piglets. J. Anim. Sci..

[29] Oliviero C., Kokkonen T., Heinonen M., Sankari S., Peltoniemi O. (2009). Feeding sows with high fibre diet around farrowing and early lactation: Impact on intestinal activity, energy balance related parameters and litter performance. Res. Vet. Sci..

[30] Oliviero C., Heinonen M., Valros A., Peltoniemi O. (2010). Environmental and sow-related factors affecting the duration of farrowing. Anim. Reprod. Sci..

[31] Feyera T., Pedersen T.F., Krogh U., Foldager L., Theil P.K. (2018). Impact of sow energy status during farrowing on farrowing kinetics, frequency of stillborn piglets, and farrowing assistance. J. Anim. Sci..

[32] Von Borell E., Hurnik J.F. (1990). Stereotypic behavior and productivity of sows. Can. J. Anim. Sci..

[33] Arey D.S., Petchey A.M., Fowler V.R. (1991). The preparturient behaviour of sows in enriched pens and the effect of pre-formed nests. Appl. Anim. Behav. Sci..

[34] Jensen P. (1993). Nest building in domestic sows: The role of external stimuli. Anim. Behav..

[35] Yun J., Valros A. (2015). Benefits of prepartum nest-building behaviour on parturition and lactation in sows - a review. Asian-Australas. J. Anim. Sci..

[36] Yun J., Swan K.-M., Farmer C., Oliviero C., Peltoniemi O., Valros A. (2014). Prepartum nest-building has an impact on postpartum nursing performance and maternal behaviour in early lactating sows. Appl. Anim. Behav. Sci..

[37] Yun J., Swan K.-M., Vienola K., Farmer C., Oliviero C., Peltoniemi O., Valros A. (2013). Nest-building in sows: Effects of farrowing housing on hormonal modulation of maternal characteristics. Appl. Anim. Behav. Sci..

[38] Hoffmann G., Bentke A., Schmidt M., Ammon C., Manteuffel C., Schön P.C. (2017). Postpartum changes in the lying behavior of sows in farrowing crates. J. Vet. Behav..

[39] Yun J., Swan K.-M., Oliviero C., Peltoniemi O., Valros A. (2015). Effects of prepartum housing environment on abnormal behaviour, the farrowing process, and interactions with circulating oxytocin in sows. Appl. Anim. Behav. Sci..

[40] D’Eath R.B., Tolkamp B.J., Kyriazakis I., Lawrence A.B. (2009). ‘Freedom from hunger’ and preventing obesity: The animal welfare implications of reducing food quantity or quality. Anim. Behav..

[41] Ijichi C.L., Collins L.M., Elwood R.W. (2013). Evidence for the role of personality in stereotypy predisposition. Anim. Behav..

[42] Li X., Li J.-h., Cui S.-q., Li S.-l., Bao J. (2017). The relationship of plr to stereotypic behaviors and neurotransmitters in sows. J. Vet. Behav..

[43] Rosvold E.M., Newberry R.C., Framstad T., Andersen I.-L. (2018). Nest-building behaviour and activity budgets of sows provided with different materials. Appl. Anim. Behav. Sci..

[44] Perez-Sanchez R.E., Ordaz-Ochoa G., Juarez-Caratachea A., Garcia-Valladares A., Ortiz-Rodriguez R. (2015). Effect of number of parity on voluntary food intake of sows during the lactation period and its impact on weaning-to-estrus interval. Thecnical note. Rev. Cient.-Fac. Cienc. Vet..

[45] Thingnes S.L., Ekker A.S., Gaustad A.H., Framstad T. (2012). Ad libitum versus step-up feeding during late lactation: The effect on feed consumption, body composition and production performance in dry fed loose housed sows. Livest. Sci..

[46] Jensen P. (1988). Maternal behaviour and mother—young interactions during lactation in free-ranging domestic pigs. Appl. Anim. Behav. Sci..

[47] Yin G., Liu H., Li X., Quan D., Bao J. (2016). Effect of farrowing environment on behaviour and physiology of primiparous sows with 35-day lactation. Int. J. Appl. Res. Vet. Med..

[48] Clarkson J.M., Dwyer D.M., Flecknell P.A., Leach M.C., Rowe C. (2018). Handling method alters the hedonic value of reward in laboratory mice. Sci. Rep..

[49] Imfeld-Mueller S., Hillmann E. (2012). Anticipation of a food ball increases short-term activity levels in growing pigs. Appl. Anim. Behav. Sci..

[50] Imfeld-Mueller S., Van Wezemael L., Stauffacher M., Gygax L., Hillmann E. (2011). Do pigs distinguish between situations of different emotional valences during anticipation?. Appl. Anim. Behav. Sci..

[51] Henzen A., Gygax L. (2018). Weak general but no specific habituation in anticipating stimuli of presumed negative and positive valence by weaned piglets. Animals.

[52] Zebunke M., Langbein J., Manteuffel G., Puppe B. (2011). Autonomic reactions indicating positive affect during acoustic reward learning in domestic pigs. Anim. Behav..

[53] Düpjan S., Schön P.-C., Puppe B., Tuchscherer A., Manteuffel G. (2008). Differential vocal responses to physical and mental stressors in domestic pigs (sus scrofa). Appl. Anim. Behav. Sci..

[54] Mahnhardt S., Brietzke J., Kanitz E., Schön P.C., Tuchscherer A., Gimsa U., Manteuffel G. (2014). Anticipation and frequency of feeding affect heart reactions in domestic pigs. J. Anim. Sci..

[55] Prunier A., Quesnel H., de Bragança M.M., Kermabon A.Y. (1996). Environmental and seasonal influences on the return-to-oestrus after weaning in primiparous sows: A review. Livest. Prod. Sci..

[56] Weaver A.C., Kelly J.M., Kind K.L., Gatford K.L., Kennaway D.J., Herde P.J., van Wettere W.H.E.J. (2013). Oocyte maturation and embryo survival in nulliparous female pigs (gilts) is improved by feeding a lupin-based high-fibre diet. Reprod. Fertil. Dev..

[57] Jarrett S., Ashworth C.J. (2018). The role of dietary fibre in pig production, with a particular emphasis on reproduction. J. Anim. Sci. Biotechnol..

[58] Noblet J., Le Goff G. (2001). Effect of dietary fibre on the energy value of feeds for pigs. Anim. Feed Sci. Technol..

[59] Poulopoulou I., Eggemann A., Moors E., Lambertz C., Gauly M. (2018). Does feeding frequency during lactation affect sows’ body condition, reproduction and production performance?. Anim. Sci. J..

